# Clinical spreading of muscle weakness in amyotrophic lateral sclerosis (ALS): a study in 910 patients

**DOI:** 10.1007/s00415-024-12408-y

**Published:** 2024-07-06

**Authors:** Albert C. Ludolph, Jennifer Dietrich, Jens Dreyhaupt, Jan Kassubek, Kelly Del Tredici, Angela Rosenbohm

**Affiliations:** 1 Department of Neurology, University of Ulm, Ulm, Germany; 2 German Center for Neurodegenerative Diseases (DZNE), Ulm, Germany; 3 Neurozentrum Biberach, Biberach, Germany; 4 Institute of Epidemiology and Medical Biometry, University of Ulm, Ulm, Germany

**Keywords:** Amyotrophic lateral sclerosis, Cortical spreading, Spreading patterns, TDP-43

## Abstract

**Background:**

Neuroanatomical staging of sporadic amyotrophic lateral sclerosis (ALS) indicates that neurodegeneration may spread corticofugally.

**Methods:**

We conducted an observational study to define the initial sites of disease onset and the clinical progression (‘spreading patterns’) of motor deficits in a cohort of 910 ALS patients in Germany.

**Results:**

Mean age of ALS onset was 59.0 ± 12.6 years for males and 61.2 ± 10.5 years for females, the mean ALSFRS-R was 35.1 ± 9.2, and 7.7% of the cohort reported a family history. Onset of motor symptoms was bulbar/upper limb in 26.8%/35.9%, the right arm initially being slightly more often affected than the left (18.5% vs.16.3%). Testing on concordance of handedness and onset in the dominant arm did not reach significance. Lower limb onset was observed in 37.3%. Unilateral limb onset patients reported horizontal spreading about three times more often than vertical spreading. 71/244 bulbar onset patients reported spreading pattern to the legs, and 17/339 lumbar onset patients reported spreading secondarily to the bulbar region.

**Discussion:**

Our results indicate that, although the phenotype of so-called ‘spinal’ or ‘intraspinal’ spreading predominated, we also observed an additional clinical spreading pattern: 29.1% of patients with bulbar onset experienced spreading clinically to the legs (vice versa in 5.0% of lumbar onset patients). For obvious neuroanatomical reasons, this pattern hardly can be explained solely by a ‘spinal’ or an ‘intraspinal’ pattern of spreading. Instead, these findings complement insights from previous clinical and clinicopathological studies supporting a cortical initiation of ALS.

## Introduction

The longitudinal pattern of neuronal damage in neurodegenerative diseases has been successfully defined by neuropathological staging efforts using molecular markers [1–6]. This includes sporadic amyotrophic lateral sclerosis (ALS) using the molecular marker phosphorylated 43-kDa TAR DNA-binding protein (pTDP-43) [7–12]. However, based on the few existing clinical observational studies, it has been debated whether the progression of clinical motor symptoms reflects a primary cortical spreading or, rather, involves primarily the spinal cord [13–15].

Other authors have debated whether cortical spreading uses the corpus callosum as a connection between cortices [16] or involves cortical hyperexcitability of hemispheres [17, 18].

We tested the hypothesis that contrary to the descriptive studies by Ravits et al. [13–15], cortical spreading of ALS is a significant part of the disease process, as suggested by Turner et al. [19] and more recently by Maranzano et al. [20]. It has been reported that handedness plays a role in the initial clinical presentation [21, 22], whereas the relation of right to left leg onset does not reveal a clear picture. Since the involvement of certain body regions also seems to alter survival [19, 23], the understanding of spreading patterns in amyotrophic lateral sclerosis is crucial for the evaluation of the disease.

Therefore, we retrospectively examined the charts of 910 ALS patients from a single center for the site of motor deficit onset and subsequent spreading of clinical symptoms by using a prespecified protocol.

## Methods

### Patient cohort

A total of 910 ALS patients from the Department of Neurology at the University of Ulm, Germany, were included between 2013 and 2020 with at least three documented regions of progression after focal symptomatic paresis (onset) in bulbar, cervical, or lumbar regions. Patients with thoracic involvement (either respiratory muscle weakness or axial trunk weakness) as well as patients with progressive muscular atrophy (PMA) were excluded. The cohort consisted of 42.0% female and 58.0% male patients. Mean age of onset was 59.0 ± 12.6 years for males and 61.2 ± 10.2 years for females. The study was performed according to the Declaration of Helsinki and approved by the local institutional ethics board (references 11/10 and 19/12). Informed written consent was obtained from all participating patients.

### Region of disease onset and progression

The region of onset and sequence of body regions affected by muscle weakness were evaluated in each patient using a standardized protocol. Clinical spreading pattern and the El Escorial criteria were recorded by a senior board-certified neurologist [24]. The consecutively affected paretic body regions (bulbar, upper limb right, upper limb left, lower limb right, lower limb left) were clinically ascertained by the neurological examination in a second or more visits with documentation of the paresis and numbered in ascending order. Isolated fasciculations or reflex abnormalities without paresis were not taken into account. Upper and lower motor neuron signs were clinically evaluated. UMN signs were defined as spasticity in any region, brisk jaw jerk and increased tendon reflexes, or Babinski response. Lower motor neuron signs were recorded as decreased reflexes, atrophy; paresis was reported on the MRC scale. Gender, age at disease onset, site of disease onset, and ALS global disease severity as measured by ALS functional rating score (ALSFRS-R) [25] were obtained. Patients with possible, probable, and definite El Escorial disease categories were included. In the case of several consecutive appointments in the outpatient clinic, the date of the first visit fulfilling the inclusion criteria was chosen for evaluation.

### Statistical analysis

Descriptive statistical analyses and visualizations were performed using Prism Version 10.1 (GraphPad, San Diego, CA, USA) and SAS, version 9.4 (SAS Institute, Cary, NC, USA). a *p* value < 0.05 was considered statistically significant. The chi-square or Fisher’s exact test as appropriate was performed to compare categorical data between groups. The binomial test of proportions was used to test the concordance between the side of upper limb onset and handedness. As a null hypothesis, the side of onset and handedness should be equally probable. Binomial test was also used for concordance of UMN with various clinical spreading forms as indicated in the text. A two-sided *p* value < 0.05 was considered statistically significant. No adjustment for multiple testing was done. All results have to be interpreted as exploratory.

## Results

The mean ALSFRS-R was 35.1 ± 9.2 points. Seventy patients (7.7%) of the cohort reported to have familial ALS and carried the following mutations: *n* = 54 with *C9ORF72* repeat expansion, *n* = 13 with *SOD1* mutations, 2 *FUS* mutations, and 1 *SCA6* mutation.

**Fig. 1:**
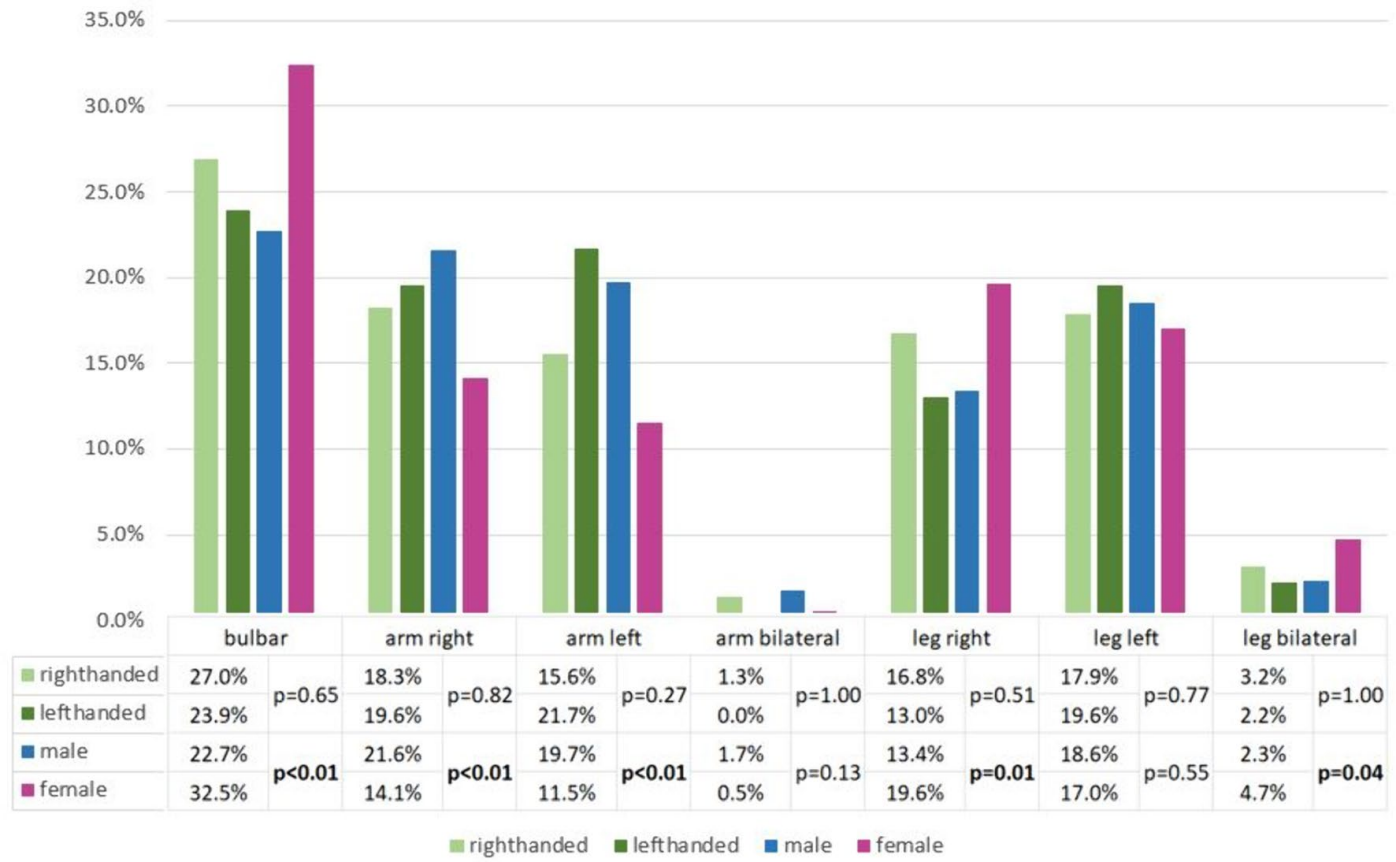
Region of onset in 910 individuals according to gender and handedness. Percentages are defined in relation to the entire group of females or males. Handedness is displayed for right and left hand dominance (*n* = 862) excluding those reporting equal dominance or missing handedness reports. Percentages were displayed in relation to the entire group of either right-handed (*n* = 816) or left-handed (*n* = 46) individuals. Chi-square tests were used for the evaluation of the association between the first symptom and handedness/gender. In the case of low numbers, Fisher’s exact test was chosen (arm bilateral and handedness/gender, leg bilateral and handedness). Significant p values are indicated in bold

Females experienced bulbar onset more frequently than males (female 32.5% vs. male 22.7%), whereas males experienced onset more often in the upper extremities (male 43.0% vs. female 26.2%), in particular the right arm (male 21.6% vs. female 14.1%) (Fig. 1). 93.3% of patients reported right-handedness, 5.3% left-handedness and 1.5% of patients were ambidextrous. Right-handed patients reported first symptoms in the bulbar region in 27.0% and 18.3% in the dominant right hand. Patients who reported left-hand dominance most frequently had a bulbar onset (23.9%) and 21.7% left-hand onset (Fig. 1).

### Region of onset

Bulbar onset of motor symptoms was reported in 26.8% of patients (*n* = 244), onset in the upper extremities was observed in 35.9% (*n* = 327), the right arm being primarily affected in 18.5% and the left arm in 16.3%. Lower limb onset was seen in 37.3% of patients (*n* = 339), with 16.0% reporting onset in the right leg, 17.9% onset in the left leg, and 3.3% recalling an onset in both lower extremities (Fig. 1).

The sequence of body regions affected with the regional spreading of muscle weakness was further analyzed in patterns depending on the site of disease onset. Further spreading patterns to other body regions are shown in Tables 1, 2 and 3. Bulbar onset patients spread mainly to the cervical region in 70.9% of patients and to the lumbar region in 29.1%. The main spreading direction after cervical onset was to the bulbar region in 27/327 (8.3%), to the contralateral arm in 210/327 (64.2%) and to the either lumbar region in 90/327 (27.5%). Ipsilateral vertical spread was reported from the right arm to the right leg in 30/327 and from the left arm to the left leg in 32/327 cases, whereas vertical contralateral spread was only—retrospectively—rarely reported (right arm to left leg 7/327, left arm to right leg in 2/327). After onset in the lumbar region, the second affected region was most frequently the contralateral leg in 216/339 patients (63.7%). The cervical region was secondarily affected in 106/339 patients (31.3%), and—surprisingly but rarely—the disease spread to the bulbar region in 17/339 patients (5.0%). Ipsilateral spread was reported from the right leg to the right arm in 30/339 and from the left leg to the left arm in 36/339 cases, whereas contralateral spread was rare (right leg to left arm 7/339, left leg to right arm in 5/339).

**Table 1:**
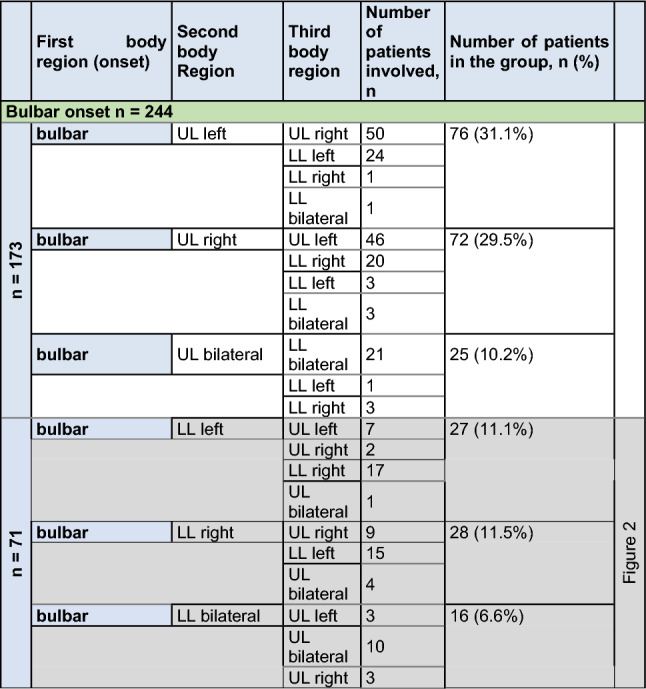
Spreading of motor symptoms from bulbar onset to the second and third affected region, as reported by patients

*UL* upper limb, *LL* lower limb

**Table 2:**
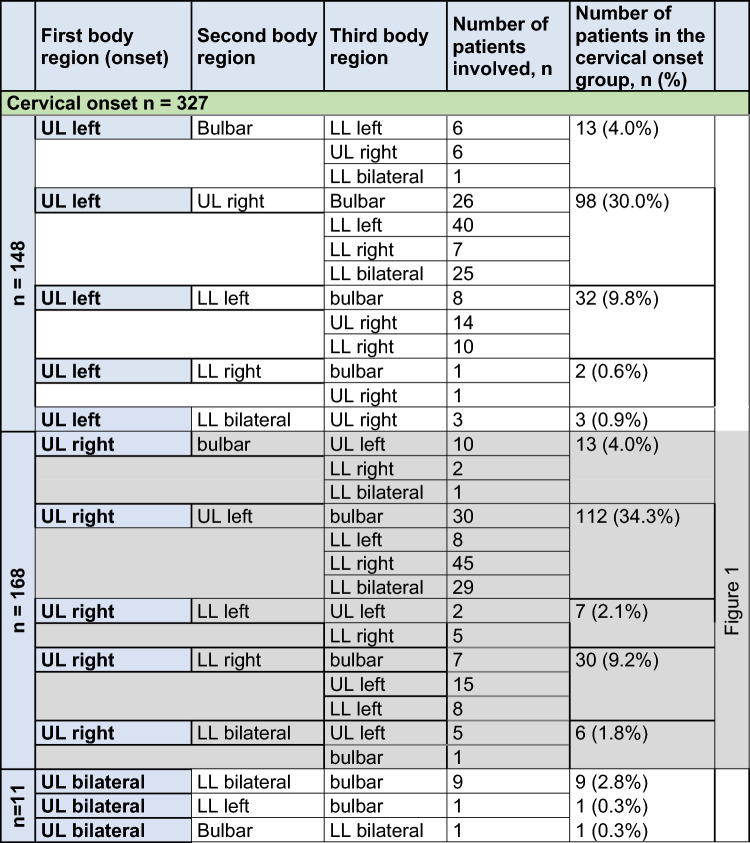
Spreading of motor symptoms from cervical onset with upper limb paresis to second and third affected region or limb, as reported by patients

*UL* upper limb, *LL* lower limb

**Table 3:**
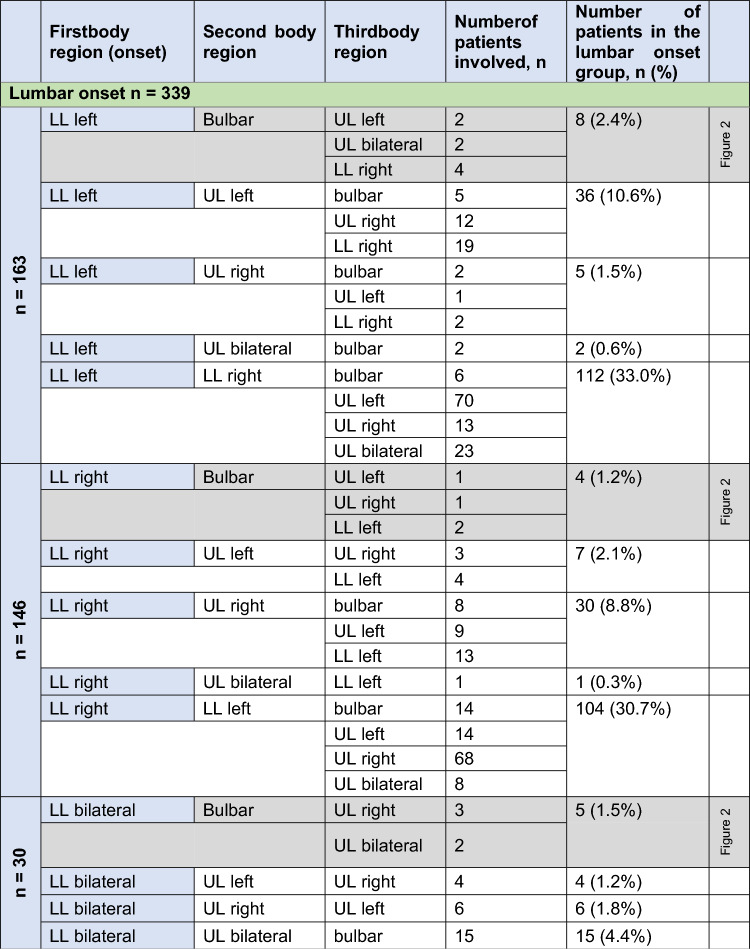
Spreading of motor symptoms from lumbar onset to the second and third affected region, as reported by patients

UL upper limb, LL lower limb

### Onset in the right upper extremity

**Fig. 2:**
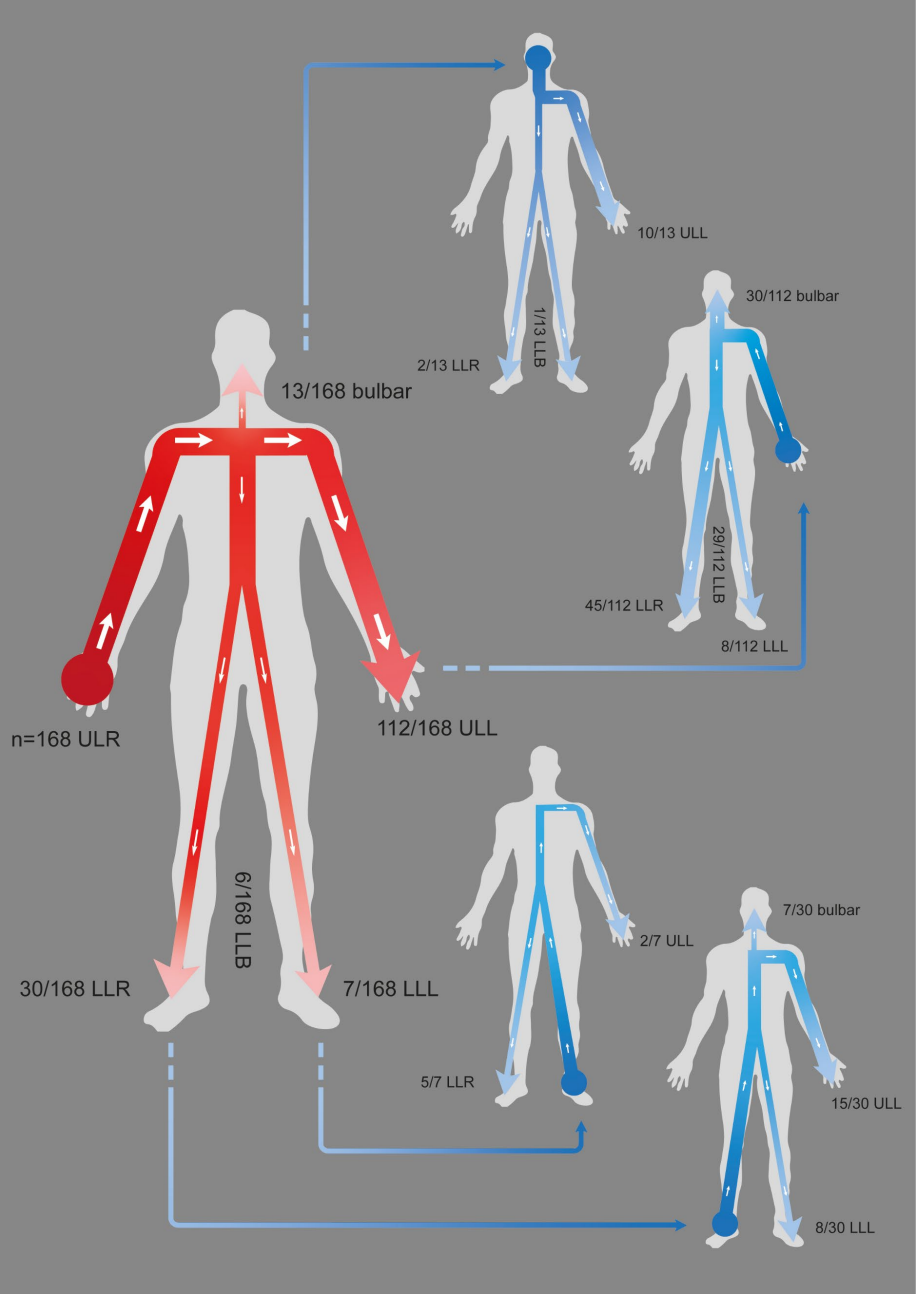
Patterns of progression after onset in the right upper extremity. *Red arrows* indicate spreading from onset in the right arm to other body regions. The arrow thickness reflects the proportion of involved cases. Body outlines with *blue arrows* represent spreading from the second involved body region to the tertiary spreading site, with arrow thickness again reflecting the proportion of involved cases. *ULR* upper limb right, *ULL* upper limb left, *LLR* lower limb right, *LLL* lower limb left, *ULB* upper limb bilateral, *LLB* lower limb bilateral

Disease onset was more frequently observed in the right (168/327; 51.4%) than in the left arm (148/327; 45.3%). Following onset in the right arm (*n* = 168), the disease secondarily spread most frequently horizontally to the left arm (*n* = 112) or vertically to the right lower extremity (*n* = 30) (Fig. 2, *body outline with red*). In the remaining patients, we observed secondary spread to bulbar muscles (*n* = 13), to both legs (6/168) and to the left leg (7/168) (Fig. 2, *body outline with red*). When the disease spread secondarily to the left upper extremity, the tertiary site affected was the right lower extremity (*n* = 45), or bulbar muscles (*n* = 30), or both legs (29/112) (Fig. 2, *blue arrows*). When the disease was reported to have spread secondarily to the right lower leg, the site tertiary affected was the left upper limb (*n* = 15), left lower limb (8/30), or bulbar muscles (7/30) (Fig. 2).

### Onset in other regions

An onset at the left upper limb showed rather similar results in the adjacent regions secondarily involved: A second site was reported in the bulbar region in 13/168 (7.7%) after onset in the left arm. A progression to the contralateral right arm was reported in 98/148 (66.2%) and a progression to the ipsilateral (left) leg in 32/148 (21.6%), mirroring with almost identical results in the right upper limb onset type: from right upper limb onset, the second body region reported was bulbar in 13/148 (8.8%), the contralateral left arm in 112/168 (66.7%), and the ipsilateral right leg in 30/168 (17.9%).

Spreading after onset in an extremity was more likely to be horizontal to the opposite limb (arms: right to left 34.3%, left to right 30.0%; legs: right to left 30.7%, left to right 30.0%) than vertical (right arm to right leg 9.2%, left arm to left leg 9.8%; right leg to right arm 8.8%, left leg to left arm 10.6%).

The influence of the side of upper limb onset and handedness was tested in the subgroup with unilateral handedness and unilateral upper limb onset by means of a binomial test of proportions. After excluding ambidextrous patients, a total of 159/295 (53.9%) reported equilateral handedness and side of upper limb onset (right 149/276, left 10/19). Concordance was not significant (*p* = 0.20).

Concordance for horizontal spreading from one arm to the contralateral arm with involvement of the UMN in the first symptomatic region was significant with *p* < 0.01 (148/210 cases; 70.5% (95% CI 63.8–6.6%)).

Regarding onset in the lumbar region, the second region again was most often the contralateral leg (from right to left in 104/146 (71.2%) and from the left to the right leg in 112/163 (68.7%). The next frequent spreading was to the ipsilateral upper limb (right leg to right arm in 30/146 (20.5%) and from left leg to left arm in 36/163 (22.1%)).

Concordance for horizontal spreading from one leg to the contralateral leg with involvement of the UMN in the first symptom region was significant with *p* < 0.01 (139/210 cases; 66.2% (59.4%-72.6%)).

### Spreading patterns following bulbar or lumbar onset

**Fig. 3:**
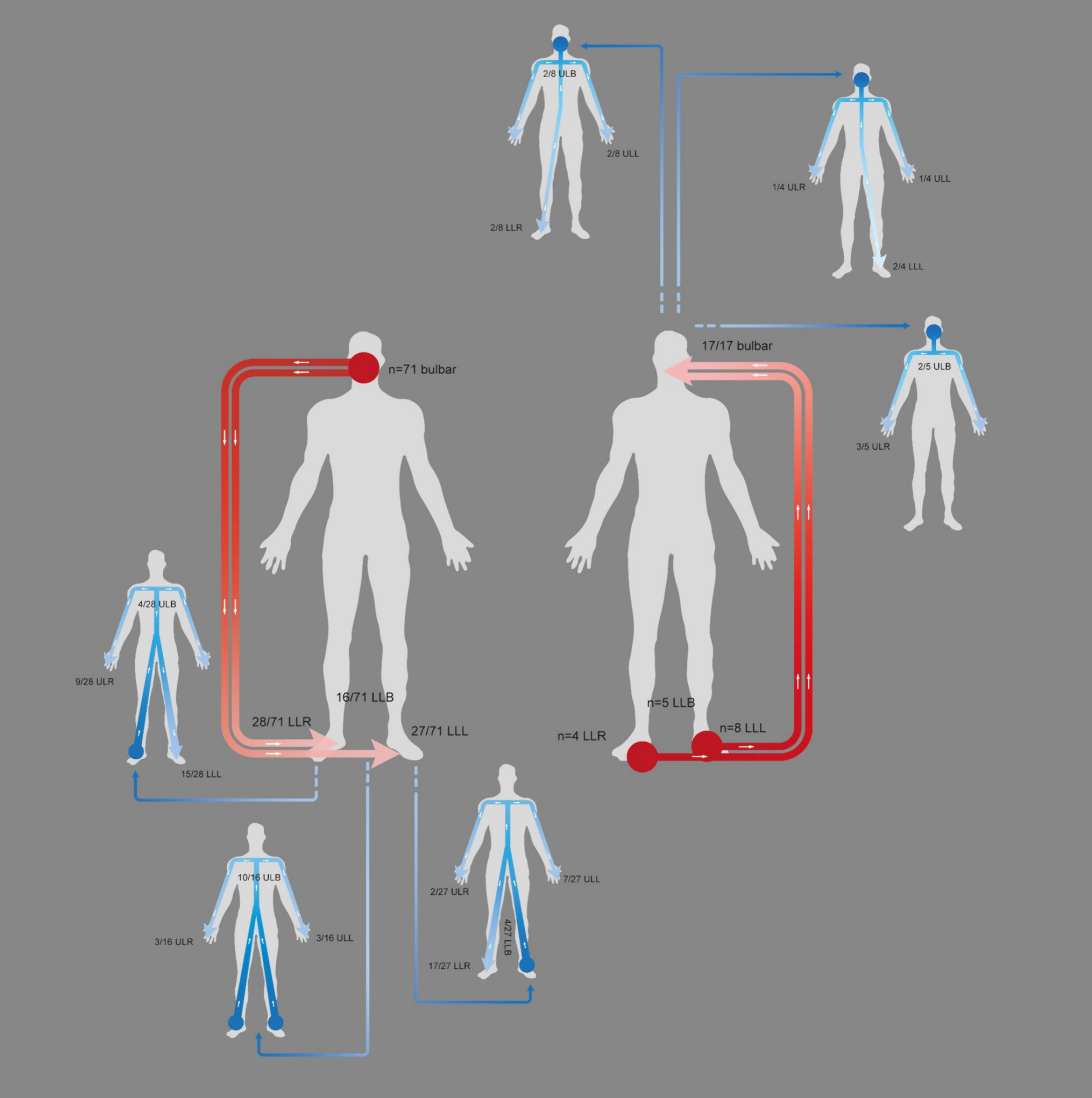
Patterns of progression after bulbar or lumbar onset. *Red arrows* reflect spreading from onset in the bulbar region to the lumbar region (left side; *n* = 71) or vice versa from lumbar to bulbar involvement (right side; *n* = 17). Body outlines with *blue arrows* represent spreading from the second involved body region to the tertiary spreading site. *ULR* upper limb right, *ULL* upper limb left, *LLR* lower limb right, *LLL* lower limb left, *ULB* upper limb bilateral, *LLB* lower limb bilateral

An exception from the continuous clinical spreading pattern either horizontally or vertically were the patients who reported spreading from the bulbar muscles to the lower extremities or vice versa. 71/244 bulbar onset patients reported spreading of symptoms to the legs, and 17/339 lumbar onset patients reported secondary spreading from the legs to the bulbar region. Spreading from bulbar onset to either of the legs (craniocaudal) was the most frequently observed pattern (Fig. 3, *red arrows*). Rostrocaudal progression was reported by 71 patients (28 to the right leg, 27 to the left leg, and 16 to both legs simultaneously). Caudorostral spreading from either leg to the bulbar muscles was reported by 17 patients (with a respective onset in the right/left/both leg(s) in *n* = 146/163/30 patients) (Fig. 3).

89.9% (95% CI 81.0–95.5%) of cases with spreading from the bulbar region to the legs and vice versa showed clinical signs of UMN involvement. The concordance between clinical UMN signs and this subgroup in the binomial test was significant (*p* < 0.01).

## Discussion

In this clinical study, we wanted to determine whether in ALS the spreading pattern of motor deficits is ‘spinal’ (or intraspinal), as proposed by Ravits et al. [13, 14, 26]. Here, we found that the spreading pattern in ALS resembled that described elsewhere [13, 14, 20, 26, 27]; however, in contrast to some of these studies, we also encountered 29.1% (71/244) of bulbar onset cases and 5.0% (17/339) of lumbar onset cases a clinical spreading pattern from bulbar muscles to the leg muscles and vice versa, which cannot be explained by a propagation of spinal cord lesions alone. Similar results were reported by Gromicho et al. [27] in a multicenter study who described the influence of UMN and LMN involvement on disease spread in a cohort of 1376 patients: Spreading from the bulbar to the lumbosacral region was observed in 35%/26% (bulbar UMN/LMN) of bulbar- onset patients. Lumbosacral to bulbar spreading was reported in 16.5% (UMN) and 12.5% (LMN). A predominance of UMN signs seemed to reduce the craniocaudal spreading time [27]. The group postulated a specific resistance of bulbar neurons to disease progression and proposed a model of independent lower and upper motor neuron degeneration modulated by the involvement of descending motor pathways.

More recently, Maranzano et al. [20] analyzed the concordance of UMN involvement and horizontal vs. vertical spreading patterns and also focused on the affection of frontal symptoms in the ECAS neuropsychological screening. In their analysis, they excluded patients with a bulbar onset. Our results were quite similar to theirs and corroborate their findings by analyzing the bulbar onset subgroup by a slightly different method.

Our study did not confirm the observations made by Turner et al. [21] in a statistically significant manner, namely, that the onset of motor symptoms in ALS is more frequent in the dominant upper extremity. In a cohort of 343 patients, they reported concordance for the side of onset and handedness (64% equilateral onset and handedness). The absence of such an effect was reported for the lower extremities. In our study, by contrast, the side of onset in the upper limbs and handedness were independent; binomial testing for concordance showed no significance but showed the same direction (*p* = 0.20; 53.9% equilateral onset and handedness).

An explanation could be the different proportions in right/left-handedness in both studies: Turner et al. [21] reported 87% right-handed vs. 10% left-handed patients, whereas only 5.3% of our cohort reported left-handedness, which, in turn, may have influenced the concordance test. Another difference could be the cohort size: the left-handed subgroups in Turner´s study were small and, accordingly, more susceptible to bias than in here.

In addition to the clinical so-called ‘spinal’ progression pattern described by Ravits et al. [13, 14], we found that “horizontal” spreading of paresis (leg to leg or arm to arm) was more frequently reported by patients than spreading from arm to leg and vice versa. Concordance between signs of UMN at the first symptom in the horizontal spreading patterns were significant in arms and legs. Moreover, presumably owing to the larger number of individuals studied, we could show that, in a significant subset (29.1%), clinical progression of paresis did not correspond to the ‘spinal’ pattern of progression. Specifically, our cohort included a large number of bulbar-onset patients in whom the paresis “jumped” to the lower extremities, while initially sparing the upper extremities. In addition, a smaller number of patients experiencing lower limb disease onset (5.0%) secondarily developed bulbar disease.

This pattern of progression from the lower extremities to the bulbar region has also been seen in other studies, e.g., Zhang et al. [28], who reported in a cohort of 196 lower limb onset patients 5.1% with spreading from the legs to the bulbar region, and 2% from the legs to the bulbar muscles and unilateral arm simultaneously. Finally, Maranzano et al. [20] also reported spreading from the bulbar region to the leg and vice versa in 9.1% of a cohort of 555 patients. Current theories on molecular mechanisms of spreading in ALS include, in addition to cell-to-cell transport deficits and conversion of a natively folded protein into a misfolded version in genetic ALS variants, the cell-to-cell transfer of pTDP43 to immediately adjacent neurons [29–31]. Relying on another neuroanatomical model, the origin of pTDP-43 pathology is thought to spread from cortical regions via corticofugal fibers monosynaptically to subcortical α-motor neurons [8, 32, 33]. Experimental arguments for this hypothesis could be generated in knockout mouse models for subcerebral projection neurons that led to reduced UMN syndromes and disease progression [34]. Thus, propagation from affected bulbar motor neurons to the lower limbs is explained by anatomical proximity that can only be found in the brain.

Maranzano et al. [35] revealed an association between clinically prominent UMN dysfunction and more significant behavioral impairment measured by the Edinburgh Cognitive and Behavioral ALS Screen and Frontal Behaviour Inventory in 110 patients, supporting the hypothesis of a spreading of pathology from the motor cortex to the ventromedial prefrontal and orbitofrontal cortex. In a follow-up study with 913 Italian ALS patients, the same group was able to show that major involvement of the motor cortex with a vertical pattern of disease progression was associated with a higher degree of disability and cognitive impairment [20].

Although patient recollection might have been limited, we regard the clinical spreading pattern from the bulbar muscles to the lower extremities as remarkable because the number of patients was substantial (*n* = 71/244) and because, for obvious neuroanatomical reasons, this pattern hardly can be explained by a purely ‘spinal’ or intraspinal pattern of disease progression. In line with our assumption of a primarily cortical influence on the disease pattern, Thakore et al. [36] could show a nonsegmental progression of arm muscle weakness for different sites of onset that cannot be explained by means of a spread of neurodegeneration within the spinal cord [36].

Subclinical signs of UMN involvement, such as DTI-based involvement of corticoefferent fibers in the corticospinal tract of pure LMN disease [37] has also been shown as well as presymptomatic cortical hyperexcitability [38]. Since evaluation in other large studies also has revealed spreading from the lumbar region to bulbar muscles and vice versa in clinically LMN onset [27], the cortical origin of ALS does not always seem to be represented as classical clinical signs of UMN involvement.

Our study has the following limitations: The original reports of the patients might have been biased and attributable to poor memory in some cases and/or influenced by the subjective experience of paresis. Memory of consecutive paresis spreading was controlled for inconsistencies in every clinical visit to reduce this potential bias. Another limitation could be a lack of clinical verification of the reported spreading pattern in patients with rapid disease progression owing to the subsequent involvement of 2 or 3 paretic body regions between two clinical visits. An obvious strength of our study is the large cohort size.

In conclusion, the current study provides support for the hypothesis raised by neuroanatomical studies that ALS may be a disease primarily of the corticomotorneuronal system [8, 32]. It accounts for all patterns of clinical spreading of paresis reported and discussed here or in earlier studies [13–15, 26, 29, 31]. The terms ‘contiguous’ (i.e., ‘adjacent’, ‘local’, ‘side-to-side’) for what has been proposed as spinal or intraspinal disease progression vs. ‘non-contiguous’ (i.e., ‘remote’, ‘end-to-end’) for corticofugal monosynaptic disease progression are insufficiently precise, and the term ‘contiguous’ is erroneous because there is currently no neuroanatomical or neuropathological evidence for spreading of pTDP-43 pathology in sporadic ALS along spinal cord α-motor neuron dendrites, α-motor neuron axon collaterals that terminate on Renshaw cells, or via the Renshaw cells themselves to the contralateral or ipsilateral side of the spinal cord. Thus, similar to the Ravits group [15], we propose—but for fundamentally different reasons—that a cortical pattern of propagation is responsible for the progression of all spreading patterns of clinical pareses in ALS. This concept needs to be further translated into revisions of the formal ALS diagnostic criteria [39].

## Conclusions

The current findings further underline the importance of understanding neuroanatomical basis of clinical disease patterns. Our findings complement insights from previous clinical and clinicopathological studies supporting a cortical initiation of ALS.

## Data Availability

Group-level outputs, post-hoc statistics and additional information on data processing can be requested from the corresponding author. Individual subject data cannot be made available due to departmental policies.
